# Probing the potential of salinity-tolerant endophytic bacteria to improve the growth of mungbean [*Vigna radiata* (L.) Wilczek]

**DOI:** 10.3389/fmicb.2023.1149004

**Published:** 2023-12-04

**Authors:** Syeda Tahseen Zahra, Mohsin Tariq, Muhammad Abdullah, Marriam Zafar, Tahira Yasmeen, Muhammad Shafiq Shahid, Haitham E. M. Zaki, Amanat Ali

**Affiliations:** ^1^Department of Bioinformatics and Biotechnology, Government College University Faisalabad, Faisalabad, Punjab, Pakistan; ^2^Department of Environmental Sciences, Government College University Faisalabad, Faisalabad, Punjab, Pakistan; ^3^Department of Plant Sciences, College of Agricultural and Marine Sciences, Sultan Qaboos University, Muscat, Oman; ^4^Horticulture Department, Faculty of Agriculture, Minia University, El-Minia, Egypt; ^5^Applied Biotechnology Department, University of Technology and Applied Sciences-Sur, Sur, Oman; ^6^Nuclear Institute of Agriculture (NIA), Tandojam, Pakistan

**Keywords:** mungbean, biofertilizer, salt-tolerance, endophytic bacteria, *Bradyrhizobium japonicum*

## Abstract

Soil salinity is one of the major limiting factors in plant growth regulation. Salinity-tolerant endophytic bacteria (STEB) can be used to alleviate the negative effects of salinity and promote plant growth. In this study, thirteen endophytic bacteria were isolated from mungbean roots and tested for NaCl salt-tolerance up to 4%. Six bacterial isolates, TMB2, TMB3, TMB5, TMB6, TMB7 and TMB9, demonstrated the ability to tolerate salt. Plant growth-promoting properties such as phosphate solubilization, indole-3-acetic acid (IAA) production, nitrogen fixation, zinc solubilization, biofilm formation and hydrolytic enzyme production were tested *in vitro* under saline conditions. Eight bacterial isolates indicated phosphate solubilization potential ranging from 5.8–17.7 μg mL^−1^, wherein TMB6 was found most efficient. Ten bacterial isolates exhibited IAA production ranging from 0.3–2.1 μg mL^−1^, where TMB7 indicated the highest potential. All the bacterial isolates except TMB13 exhibited nitrogenase activity. Three isolates, TMB6, TMB7 and TMB9, were able to solubilize zinc on tris-minimal media. All isolates were capable of forming biofilm except TMB12 and TMB13. Only TMB2, TMB6 and TMB7 exhibited cellulase activity, while TMB2 and TMB7 exhibited pectinase production. Based on *in vitro* testing, six efficient STEB were selected and subjected to the further studies. *16S rRNA* gene sequencing of efficient STEB revealed the maximum similarity between TMB2 and *Rhizobium pusense*, TMB3 and *Agrobacterium leguminum*, TMB5 and *Achromobacter denitrificans*, TMB6 and *Pseudomonas extremorientalis*, TMB7 and *Bradyrhizobium japonicum* and TMB9 and *Serratia quinivorans*. This is the first international report on the existence of *A. leguminum*, *A. denitrificans*, *P. extremorientalis* and *S. quinivorans* inside the roots of mungbean. Under controlled-conditions, inoculation of *P. extremorientalis* TMB6, *B. japonicum* TMB7 and *S. quinivorans* TMB9 exhibited maximum potential to increase plant growth parameters; specifically plant dry weight was increased by up to 52%, 61% and 45%, respectively. Inoculation of *B. japonicum* TMB7 displayed the highest potential to increase plant proline, glycine betaine and total soluble proteins contents by 77%, 78% and 64%, respectively, compared to control under saline conditions. It is suggested that the efficient STEB could be used as biofertilizers for mungbean crop productivity under saline conditions after field-testing.

## Introduction

Mungbean [*Vigna radiata* (L.) Wilczek] is a highly nutritious food and considered as the most important pulse crop worldwide. It is preferred in our daily diet due to the presence of sulfur comprising amino acids and high phosphorus content. The global mungbean cultivated area is approximately 7.3 million hectares with an average yield of 721 kg ha^−1^ (Nair and Schreinemachers, 2020). This crop has a strategic position in Asian countries for its nutritional security, being rich in carbohydrates, proteins, vitamins and minerals. In Pakistan, the total area under cultivation of mungbean is approximately 302,000 hectare with a production of 264,000 tonne (Javed et al., 2021; Economic Survey of Pakistan, 2021–2022). Mungbean seeds are highly nutritious, containing 59–65% carbohydrates, 24–28% proteins, 3.5–4.5% fibers, 1–1.5% fats and 334–344 kcal energy (Sehrawat et al., 2021). Mungbean is used as a staple food in different Asian countries including Pakistan, Thailand, India and the Philippines (Delic et al., 2009).

The production of legume grains retards due to numerous abiotic stresses, particularly salt stress, which impairs the activity of symbiotic bacteria and reduces the plant growth (Pataczek et al., 2018; Kartik et al., 2021). Salinity negatively impacts plant physiological activities by plant dehydration, disrupting ionic and osmotic balance, which ultimately causes plant death (Shahzad et al., 2017; Majeed and Muhammad, 2019). Mungbean is highly sensitive towards salinity with a threshold level of electrical conductivity (EC) of 1.8 dS m^−1^ (Pataczek et al., 2018). Plants adopt different strategies such as antioxidant synthesis, osmosensing and maintaining the ionic-homeostasis to cope with salt stress (Chauhan et al., 2022). Ecofriendly salt-tolerant plant growth-promoting bacteria (PGPB) are promiscuous to improve these mechanisms of plants to tolerate salinity.

The agriculture sector largely relies on the synthetic fertilizers, specifically urea and diammonium phosphate (Economic Survey of Pakistan, 2021–2022). Chemical fertilizers are made up of salts of nitrate, ammonium, phosphorus, and potassium, as well as a variety of heavy metals and regular nucleosides (Sabry, 2015). Chemical fertilizer use has increased dramatically in recent years. Careless use of chemical fertilizer results in the accumulation of heavy metals in plant structures, which then infiltrate our food chain (Savci, 2012; Alsafran et al., 2022). It can pollute our environment by contaminating water, soil and air, which entails huge environmental costs and pose serious threats to human health. Extensive use of chemical fertilizers has distorted the nitrogen cycle and other biological processes; prompting global concerns about increased emission of nitrogen oxides, soil acidification and water eutrophication (Fox et al., 2007; Conway and Pretty, 2013; Singh et al., 2019). Widespread application of fertilizers, urbanization, large scale farming and improper farming practices are some of the major causes of soil salinity. The soil salinization is increasing day by day and contaminates agricultural land (Upadhyay and Chauhan, 2022). Alternative methods are required to meet the food demand in a sustainable manner.

Biofertilizers are environment-friendly alternatives to chemical fertilizers. Biofertilizers contain PGPB, which can be applied to the soil or seed surfaces to promote plant growth by improving nutrient availability to plants and controlling phytopathogens (Agri et al., 2022; Valle-Romero et al., 2023). Biofertilizers are host specific, so the nutrients provided by them are less prone to leaching and volatilization, making them ideal for sustainable agriculture (Bhardwaj et al., 2014; Simarmata et al., 2016; Imran et al., 2021). PGPB improve plant growth directly by a variety of mechanisms, primarily including nitrogen fixation, phosphate solubilization and phytohormone production; and indirectly by bioantagonism and inducing systemic resistance (Upadhyay et al., 2022). These beneficial bacteria are mostly present in the plant rhizosphere, root interior and inside nodules. Efficiency of biofertilizers reduces due to the salt stress, as salinity impairs the bacterial cell metabolism and reduces the production of plant growth-promoting substances (Deshwal and Kumar, 2013). The high salt concentration adversely affects the important processes such as decomposition, nitrification, denitrification, soil biodiversity and microbial activity (Kumawat et al., 2022). Salt-tolerant PGPB produce phytostimulants, plant defense-related enzymes including catalases, superoxide dismutases, peroxidases and glucanases, upregulate the expression of Na^+^/K^+^ ion channel proteins, which helps to maintain ionic homeostasis and increase plant growth (Chauhan and Upadhyay, 2023; Singh et al., 2023).

Endophytic bacteria have magnanimous potential to promote plant growth, since they live in the closer proximity or inside the plant (Chanway et al., 2000; Dalal and Kulkarni, 2013; Afzal et al., 2019). They are better protected from the challenging environment as they invade plant roots and reside in the root cortical region (Compant et al., 2005; Ryan et al., 2008; Zhang et al., 2020). Endophytic bacteria have a better ability to symbiotically associate with their host plants compared to rhizospheric bacteria (Bacilio-Jiménez et al., 2003). Endophytes regulate plant defense mechanisms by producing antioxidants and to mitigate the oxidative damage caused by salt stress and help plants to tolerate the stress (Jhuma et al., 2021; Chaudhary et al., 2022; Kamran et al., 2022). Moreover, they upregulate the expression of SOS1 Na^+^/ K^+^ antiporter which control the Na^+^ and K^+^ efflux to maintain ionic-homeostasis inside the plant cell (Tyagi et al., 2022). Several endophytic bacteria well-known for improving plant growth are *Burkholderia, Herbaspirillum, Pantoea, Gluconobacter, Klebsiella, Rahnella, Pseudomonas, Bacillus, Xanthomonas, Stenotrophomonas,* *Variovorax* (Riggs et al., 2001; Rosenblueth and Martínez-Romero, 2006; Doty et al., 2009; Rat et al., 2021).

In this study, salinity-tolerant endophytic bacteria (STEB) were isolated from the mungbean root and characterized *in vitro* for the plant growth-promoting properties under saline conditions. Potential bacteria from the *in vitro* testing were phylogenetically identified by *16S rRNA* gene sequence analysis and evaluated under controlled-conditions for plant growth-promoting properties under saline conditions.

## Materials and methods

### Sample collection and isolation of endophytic bacteria

A 8-week-old Mungbean [*Vigna radiata* (L.) Wilczek] plants were collected from the cultivation site of Government College University Faisalabad, Pakistan (GPS coordinates at 31°23′42.5″ N and 73°01′45.5″ E). Intact roots were washed with water, and surface sterilized by dipping in 5% bleach for 2 min and 70% ethanol for 30 s. Roots were washed with sterilized water to remove the effect of chemicals. One-gram roots were separated from the plants using sterilized forceps and crushed in a sterilized mortar pestle within 3 mL saline solution (0.85% NaCl). Each root suspension was serially diluted up to 10^−5^ dilution. An aliquot of 100 μL from each dilution was spread on yeast extract mannitol (YEM) plates and incubated at 28 ± 2°C for 48 h (Shahid et al., 2015; Tsegaye et al., 2019). Bacterial colonies showing different morphology were selected and purified by sub-culturing (Adamu-Governor et al., 2018). Size and shape of bacterial cells were observed under light microscope. Gram’s reaction was also performed according to Wang et al. (2017).

### Screening of salt-tolerant endophytic bacteria

Salt-tolerance ability of isolated bacteria was evaluated according to Verma et al. (2020), at varying levels of NaCl concentrations. YEM broth (20 mL) in a 50 mL flask was prepared containing different concentrations of NaCl, i.e., 0.5, 0.1, 1.5, 2, 3 and 4% (w/v). Bacterial culture (0.1 mL) was inoculated in each flask and incubated at 28 ± 2°C for 42 h. Bacterial culture without salt was used as control. Optical density (OD) of bacterial growth was recorded after every 6 h at 600 nm using spectrophotometer (Patil et al., 2014).

### Phosphate solubilization

Screening of phosphate solubilizing bacteria was performed according to Oo et al. (2020) with some modifications. A single colony of bacterial isolate was spotted on Pikovskaya’s agar plate supplemented with 2% NaCl (w/v) and incubated at 28 ± 2°C for 7 days. Halo zone formation was observed around colonies to identify phosphate solubilization potential (Linu et al., 2019; Nacoon et al., 2020). Phosphate solubilization was quantified by the Phospho-molybdate blue color method according to Khan et al. (2022). Bacterial cultures were grown in Pikovskaya’s broth supplemented with 2% NaCl (w/v) and incubated at 28 ± 2°C for 7 days. After incubation, bacterial cultures were centrifuged for 10 min at 13,000 rpm and 1 mL of supernatant was mixed with 0.2 mL Phospho-molybdate regent, blue color production was observed, and absorbance was recorded at 882 nm using spectrophotometer. A phosphate standards curve was prepared to quantify phosphate concentration of samples (Behera et al., 2017).

### Indole-3-acetic acid production

Indole-3-acetic acid (IAA) production of bacterial isolates was determined by Salkowski’s calorimetric assay. Bacterial cultures were grown in YEM broth, supplemented with L-tryptophan (100 μg mL^−1^) and 2% NaCl (w/v), incubated at 28 ± 2°C for 48 h and centrifuged at 12,000 rpm for 10 min. Salkowski’s reagent (4 mL) was mixed with 1 mL of supernatant, gently mixed and incubated for 30 min at room temperature (Bhattacharyya et al., 2020). Pink coloration was taken as indication of IAA production and its absorbance was measured at 530 nm using spectrophotometer. An IAA standards curve was prepared to quantify IAA concentration of samples (Myo et al., 2019; Hyder et al., 2020).

### Nitrogen fixation

The ability of bacterial isolates to fix nitrogen was tested by inoculating a single colony on solid nitrogen free media [containing (g L^−1^) mannitol 20 g, K_2_HPO_4_ 0.2 g, NaCl 0.2 g, MgSO_4_ 0.2 g, K_2_SO_4_ 0.1 g, CaCO_3_ 5.0 g, agar 20 g] supplemented with 2% NaCl (w/v) and incubated at 28 ± 2°C for 48 h. After incubation, nitrogen fixation was determined based on the bacterial growth and recorded as arbitrary values weak (+), moderate (++), strong (+++) or negative (−) (Hardarson and Danso, 1993; Mirza and Rodrigues, 2012).

### Zinc mobilization

*In vitro* qualitative screening of zinc solubilizing bacterial isolates was measured by adopting the protocol of Ramesh et al. (2014), with some modifications. Tris-minimal agar (TMA) medium supplemented with 2% NaCl (w/v), containing insoluble 0.1% zinc source, i.e., ZnO and ZnCO_3_, separately. Supplemented TMA plates were spot inoculated with freshly grown bacterial cultures and incubated in the dark at 28 ± 2°C for 7 days. Halo zone formation around the bacterial colony was observed and zinc solubilization efficiency (ZSE) was calculated according to the formula (Rezaeiniko et al., 2022; Upadhayay et al., 2022).


$$ZSE=\left(\begin{array}{l} \mathrm{diameter}\;\mathrm{of}\;\mathrm{solubilization}\;\mathrm{halo}/\\ \mathrm{diameter}\;\mathrm{of}\;\mathrm{the}\;\mathrm{colony} \end{array}\right)\times 100.$$

### Cellulase and pectinase activity assay

Cellulolytic activity of bacterial isolates was assessed by spot inoculating individual colonies onto carboxymethyl cellulose (10 g L^−1^) agar plates supplemented with 2% NaCl (w/v) and incubated at 28 ± 2°C for 3 days (Islam and Roy, 2018). Plates were stained with 0.2% Congo red dye for 15 min and washed with distilled water. The appearance of a halo zone around the colony indicates cellulase activity of bacteria (Suárez-Moreno et al., 2019).

Pectinase production was determined by inoculating bacterial colonies onto pectin (10 g L^−1^) agar plates, supplemented with 2% NaCl (w/v) and incubated at 28 ± 2°C for 7 days (Devi et al., 2022). Plates were stained with 1% iodine solution for 15 min and washed with distilled water. The formation of a halo zone around the colony indicated pectinase activity of bacterial cultures (Tsegaye et al., 2019).

### Biofilm formation assay

Biofilm formation by bacterial isolates was tested according to Zahra et al. (2023), by using a microtiter plate. Bacterial isolates were grown up to an optical density (OD_600 nm_) of 2 in YEM broth medium, supplemented with 2% NaCl (w/v). Bacterial cultures were centrifuged at 6,000 rpm for 2 min. The supernatant was discarded and the pellet was washed with sterile water. The bacterial cells were resuspended in fresh YEM broth and diluted to an OD_600 nm_ of 0.2. An aliquot (150 μL) of each bacterial cell suspension was added to 96-well polyvinyl chloride (PVC) plate in six replicates and incubated at 28°C for 48 h. After incubation, each bacterial culture was removed from wells and gently washed with sterilized water. Wells were stained with 150 μL of crystal violet (0.001%) for 15 min. After staining, crystal violet was removed, and wells were washed with sterilized water and air dried. Crystal violet dye absorbed by the wells was solubilized by adding 150 μL of 95% ethanol. Biofilm formation was quantified by measuring the amount of absorbed dye at OD_570 nm_ in a microtiter plate reader (Sampedro et al., 2020; Jhuma et al., 2021).

### Microbial compatibility assay

Microbial compatibility tests of bacterial isolates were assessed according to Tariq et al. (2014) by pour plate technique to determine the compatibility of isolates with each other. In this assay, a pairs of bacterial isolates was designated as A and B, and checked for compatibility. Log phase grown culture of isolate A was diluted to 10^4^ cfu mL^−1^ and 3 mL of the culture was mixed in 25 mL hand-cool molten nutrient agar medium. It was poured into Petri plates and incubated at 28 ± 2°C for 24 h. Concentrated culture (3 μL) of isolate B was inoculated in the center of the plate and incubated at 28 ± 2°C for 48 h. The zone of inhibition that developed around bacterial isolate B was recorded. The appearance of a zone of inhibition represented that the bacterial pair was not compatible with each other, represented by a red box. If no zone of inhibition appeared, the bacterial pair was considered compatible and represented by a green box. Each isolate pair was tested in this manner, and compatibility and non-compatibility was presented as green and red box, respectively (Zul et al., 2022).

### Molecular identification and phylogenetic analysis of efficient endophytic bacteria

Six efficient STEB were identified phylogenetically by sequencing the *16S rRNA* gene, according to La Pierre et al. (2017). The *16S rRNA* gene was amplified using universal primers fD1 (5′-AGAGTTTGATCCTGGCTCAG-3′) and rD1 (5′-AAGGAGGTGATCCAGCC-3′) (Weisburg et al., 1991). A 25 μL reaction mixture was prepared for *16S rRNA* gene amplification by using the PCR recipe [10X Taq polymerase buffer 2.5 μL, 2 mM dNTPs 2.5 μL, (10 pmoles 100 μL^−1^) primers fD1 & rD1 2 μL, 25 mM MgCl 2 μL, (5 U μL) Taq polymerase enzyme 0.3 μL, H_2_O 11.7 μL, (20 ng μL^−1^) template DNA 2 μL]. The reaction mixture was placed in a thermocycler for amplification and adjusted initial denaturation to 5 min at 94°C, followed by 30 cycles of denaturation at 94°C for 60 s, primer annealing at 55°C for 50 s, primer extension at 72°C for 1 min 40 s and final extension at 72°C for 5 min. After amplification, the amplicons were examined in a gel documentation system on 1% agarose gel. The amplified products were purified through ThermoScientific GeneJET PCR Purification Kit and Sanger sequenced using the commercial service of Macrogen, Korea. Forward and reverse sequences were assembled manually and compared with database sequences by using NCBI BLAST tool (Altschul, 1990). Closely related authentic sequences were retrieved from databases, and pairwise sequence comparisons were performed using Sequence Demarcation Tool (SDT) v.1.2 (Zhang et al., 2000; Muhire et al., 2014). A phylogenetic tree was constructed using the maximum likelihood method as implemented by MEGA 11 with 1,000 bootstrap values (Kumar et al., 2016; Noori et al., 2021).

### Controlled-conditions experiment and biochemical analysis

The controlled-condition experiment was conducted on mungbean cultivar NM-2021 with eight treatments (TMB2, TMB3, TMB5, TMB6, TMB7, TMB9, consortia and water as control) in completely randomized design (CRD) with four replicates. Freshly grown bacterial culture was centrifuged (6,000 rpm) and bacterial pellet was resuspended in sterilized water adjust OD 0.5 (Mishra et al., 2009). Seeds were surface sterilized with 5% bleach for 2 min and washed with sterilized water. Surface-sterilized seeds were placed on Petri plates containing moist filter paper and incubated at 25 ± 2°C in a dark room for 2 days. Uniformly sized seedlings were transferred into pots containing sterilized soil, supplemented with 1% NaCl. Plants were placed in a growth chamber at 35 ± 2°C during the day and 25 ± 2°C at night. Bacterial culture (100 μL) of each treatment was applied to the roots of each plant. Plants were watered with 10 mL of quarter-strength nitrogen-free Hoagland’s solution and sterilized water on alternating days. Plants were harvested after 6 weeks of germination and agronomical parameters including root length, shoot length, plant fresh weight, plant dry weight and number of nodules per plant were recorded (Tounsi-Hammami et al., 2022). The agronomical data was statistically analyzed using CoStat window version software (Cardinali and Nason, 2013).

#### Proline contents

Proline contents were determined according to Bates et al. (1973) with some modifications. Leaf samples (0.5 g) were ground in liquid nitrogen and 10 mL chilled K-P buffer was added. The mixture was centrifuged at 13,000 rpm for 5 min. Supernatant (0.5 mL) was transferred in a test tube containing 1 mL of 3% sulphosalicylic acid and incubated at 95°C for 5 min in a water bath. After incubation, the mixture was cooled down at room temperature and 1 mL of glacial acetic acid and ninhydrin was gently added, mixed and incubated at 95°C for 20 min in a water bath. The mixture was immediately cooled down on ice. Toluene (2 mL) was added in the mixture, vortexed and incubated at room temperature for 20 min. After incubation, two layers were developed. The upper layer was carefully collected and absorbance was recorded at 520 nm using spectrophotometer. The proline contents were measured by comparing the absorbance with standard curve (Sapre et al., 2022).

#### Total soluble proteins

Total soluble proteins were quantified according to Bradford (1976) modified method. Leaf samples (0.5 g) were ground in chilled K-P buffer. After grinding, the mixture was centrifuged at 13,000 rpm for 5 min. Supernatant (0.1 mL) was collected, Bradford reagent (1 mL) was added and incubated at room temperature for 30 min in dark. After incubation, absorbance was recorded at 595 nm using spectrophotometer. Total soluble proteins were measured by comparing the absorbance with standard curve.

#### Glycine betaine

Glycine betaine in leaf tissues was estimated by following the modified protocol of Nawaz and Wang (2020). Fresh leaf samples (0.5 g) were ground in 10 mL chilled K-P buffer, vortexed and centrifuged at 13,000 rpm for 5 min. Supernatant (0.5 mL) was collected in a separate test tube and 1 mL of H_2_SO_4_ was added. KI_3_ (0.2 mL) was added into the reaction mixture and incubated at −4°C for 90 min. After incubation, 2.8 mL chilled dH_2_O and 6 mL of 1–2 dichloroethane was added into the mixture and incubated at room temperature for 30 min. Two layers were formed. The lower layer of red color was collected carefully, and absorbance was measured at 365 nm using a spectrophotometer. Quantify of glycine betaine contents was measured by comparing the absorbance with the standard curve.

## Results and discussion

### Isolation of endophytic bacteria

Bacterial colonies were observed on the plates after incubation. Based on colony size, shape, color, edges, surface and gum production, thirteen bacterial morphotypes were selected. Cell morphology of all bacterial isolates was rod shaped, except TMB4, TMB8 and TMB10, which showed circular cell shape. Only four bacterial isolates, TMB1, TMB4, TMB8 and TMB12, were Gram’s positive, while the rest of the bacteria were Gram’s negative (Table 1). Legume roots contain a large array of endophytic bacteria, which may play an important role in plant growth promotion directly and indirectly (Bhutani et al., 2018a). Our results are in agreement with several studies that confirmed the occurrence of bacteria in the legume root samples. Chaudhary et al. (2021) isolated *Rhizobium pusense* from the roots of mungbean and evaluated its plant growth-promoting properties. Bhutani et al. (2021) also isolated endophytic bacteria from surface sterilized roots of mungbean that demonstrated high potential to improve plant growth. Abedinzadeh et al. (2019) also isolated endophytic bacteria from roots of maize and reported that these bacteria have the ability to tolerate salinity and increase plant growth. Similarly, Hung and Annapurna (2004) also isolated 65 endophytic bacteria from soybean root and nodules.

**Table 1:** Colony and cell morphology of mungbean root endophytic bacteria.

Isolate	Colony morphology	Cell morphology	Gum production	Gram staining
TMB1	Medium, double ringed, light pink, smooth, flat	Small rod	+	+
TMB2	Large, circular, milky white, smooth, flat	Rod	+	−
TMB3	Very small, circular, white, smooth, flat	Rod	+	−
TMB4	Very small, circular, pink, wavy, flat	Circular	−	+
TMB5	Small, circular, light yellowish, smooth, flat	Small rod	+	−
TMB6	Large, irregular, yellowish, wavy, flat	Rod	+	−
TMB7	Small, circular, light pink, smooth, flat	Small rod	+	−
TMB8	Medium, circular, milky white, wavy, flat	Circular	+	+
TMB9	Small, circular, pink, wavy, flat	Rod	−	−
TMB10	Small, irregular, white, wavy, flat	Circular	+	−
TMB11	Small, circular, milky white, smooth, flat	Rod	−	−
TMB12	Very small, circular, white, smooth, flat	Small rod	−	+
TMB13	Very small, circular, yellowish white, smooth, flat	Rod	+	−

### Screening of salt-tolerant endophytic bacteria

**Figure 1:**
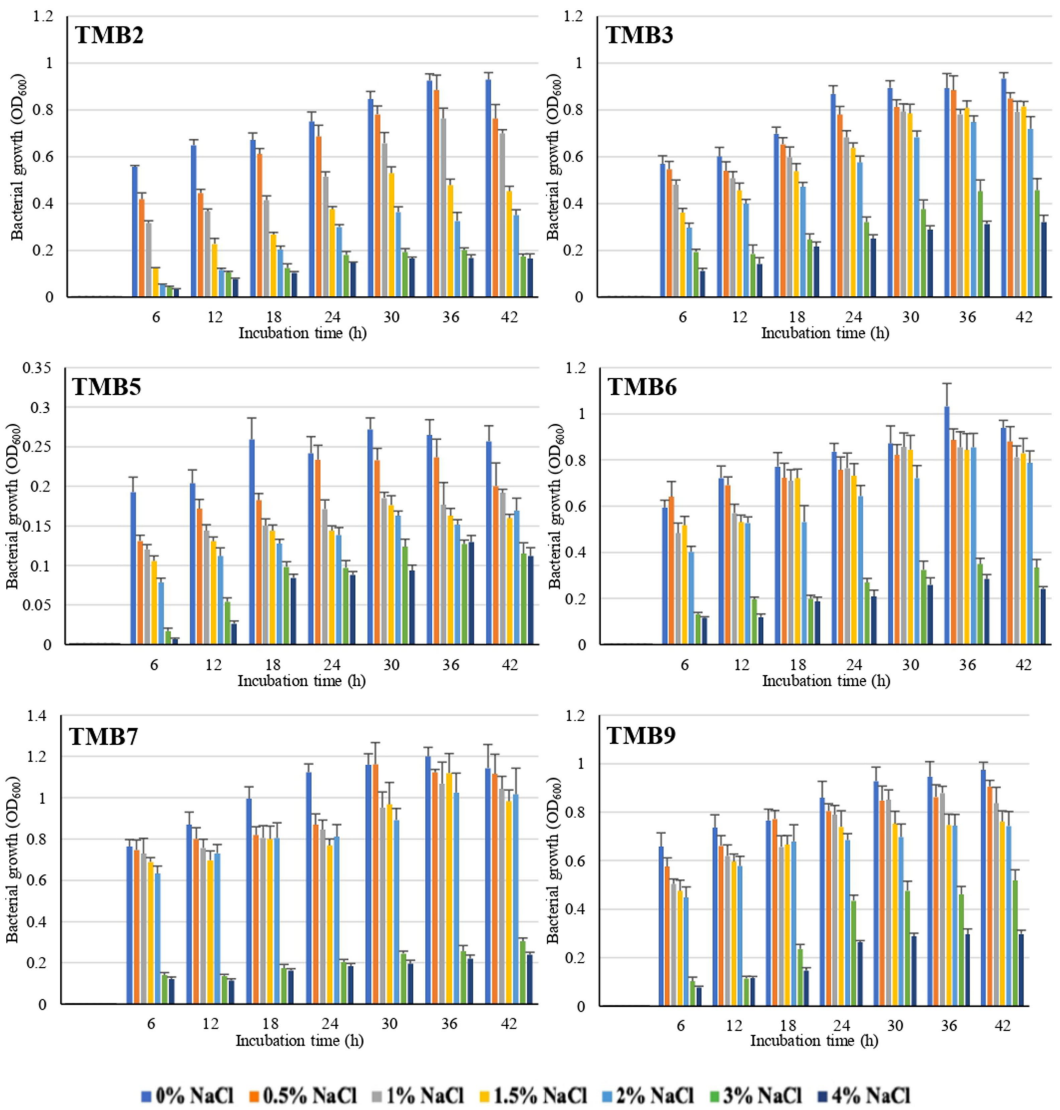
Graphical representation of mungbean root endophytic bacteria to tolerate salinity at different salt concentrations. Six bacterial isolates, TMB2, TMB3, TMB5, TMB6, TMB7 and TMB9, displayed salt-tolerance and grew well upto 2% NaCl. Growth readings of all bacterial isolates under saline conditions are mentioned in the Supplementary material.

Salinity tolerance was examined in mungbean isolates at different NaCl concentrations ranging from 0.5–4%. There was significant inhibition in bacterial growth at 3 and 4% NaCl concentration. Six bacterial isolates, TMB2, TMB3, TMB5, TMB6, TMB7 and TMB9, were able to tolerate salinity level up to 2% NaCl concentration (Figure 1), whereas three isolates, TMB1, TMB8 and TMB10, showed minor growth inhibition at 2% NaCl. The remaining four isolates, TMB4, TMB11, TMB12 and TMB13, showed significant growth inhibition at 2% NaCl salinity concentration (Supplementary Table S1). Salinity is one of the major problems for crop productivity in Pakistan due to the presence of salt contents in the soil and water (Vaishnav et al., 2019; Kartik et al., 2021). Adaptability of the bacterial inoculants to the stressed environment of any cultivation region is considered as a promiscuous feature for its use as biofertilizers (Pérez-Rodriguez et al., 2020). Recently, Kumar et al. (2021) screened salt tolerant bacteria at different concentrations of NaCl and further characterized them for plant growth promotion. Khan et al. (2015) also isolated rhizospheric and endophytic bacteria, and reported that these bacterial isolates tolerate higher concentrations of NaCl. Salinity affected soil is defined as a soil that has electrical conductivity (EC) value greater than 4 dS m^−1^ (Munns and James, 2003). EC value of 4 dS m^−1^ is equal to 0.22% NaCl concentration and EC value of 25.8 dS m^−1^ is equal to 2% NaCl concentration (observation during lab general experiments). Therefore, the potential endophytic bacteria exhibited salt tolerance ability up to 2% NaCl concentration were considered potential candidates for their use as biofertilizers at salinity affected soils. 2% NaCl is the highest realistic-concentration of salt to test microbes for salinity-tolerance, as it is the maximum concentration reported at most of the salinized land worldwide. The world’s well-known saline sites including Solonchaks (Russia), Halosols (China) and Salida (United States) have an EC ranging 8–15 dS m^−1^ (Egamberdieva et al., 2019). Soil and irrigation water in Pakistan generally have high soluble salt contents, which is a major limiting factor for plant growth. The value of EC in heavily salt affected soil of Pakistan at Uchhali Lake in the Salt Range region is 15.42 dS m^−1^, which is equal to 1.2% NaCl (Hameed et al., 2009). A concentration higher than 2% salinity is very stringent to test bacterial salt-tolerance and might result in losing too many potential bacteria. Cortés-Lorenzo et al. (2015) demonstrated that higher levels of salinity inhibited the nitrification process of nitrite-oxidizing bacteria. Hong et al. (2013) also reported that higher salinity can reduce the metabolic activity of microorganisms, results in bacterial growth inhibition and cell death. As the tested bacteria of the current study demonstrated salinity-tolerance upto 2% NaCl concentration, the beneficial characteristics of these bacteria may remain unaffected even in the saline environment. Such bacteria are promising to be used as biofertilizers for crop production at salinity affected soil and the farmland irrigated with saline-water. It is strongly suggested that biofertilizer bacteria should be tested for salt stress tolerances before application, as most of the irrigation water and soils are affected with high concentration of salts.

### Characterization of STEB for plant growth-promoting properties

Phosphate solubilization was examined in mungbean isolates under saline conditions. Out of 13 mungbean isolates, TMB2, TMB3, TMB5, TMB6, TMB7, TMB8, TMB9 and TMB10, showed phosphate solubilization ranging from 5.8–17.7 μg mL^−1^. TMB6 exhibited the highest phosphate solubilization ability, whereas TMB3 exhibited the lowest phosphate solubilization ability (Table 2). Phosphate is one of the most crucial nutrients for balanced plant growth. Deficiency of phosphate in plants usually results in stunted growth of plants (Lun et al., 2018). Previously, Hakim et al. (2020) isolated endophytic bacteria from mungbean and explained the phosphate solubilizing potential of these bacteria upto 195 μg mL^−1^. Recently, Belkebla et al. (2022) demonstrated that halotolerant PGPB isolated from south of Algeria exhibit phosphate solubilizing potential and improve wheat growth. Mahdi et al. (2021) also reported that halotolerant endophytic bacteria have the ability to solubilize phosphate and promote seed germination. Likewise, Mei et al. (2021) also demonstrated that endophytic bacteria have the potential to solubilize phosphate and their application resulted in increased pepper and tomato growth.

**Table 2:** *In vitro* testing of plant growth-promoting attributes of mungbean root endophytic bacteria.

Isolates	Phosphate Solubilization (μg mL^−1^)	IAA Production (μg mL^−1^)	Nitrogen fixation	Zinc solubilization efficiency (ZnO)	Zinc solubilization efficiency (ZnCO_3_)	Cellulase activity index	Pectinase activity index	Biofilm formation (OD_570nm_)
TMB1	0	6.8 ± 0.4	+	0	0	0	0	0.11 ± 0.012
TMB2	8.1 ± 0.44	1.1 ± 0.18	+++	0	0	1.66	1.2	0.53 ± 0.049
TMB3	5.8 ± 0.32	3.3 ± 0.28	++	0	0	0	0	1.59 ± 0.043
TMB4	0	0	+	0	0	0	0	0.63 ± 0.039
TMB5	7.9 ± 0.46	1 ± 0.19	++	0	0	0	0	0.91 ± 0.021
TMB6	17.7 ± 0.62	1.4 ± 0.33	++	260%	200%	1.75	0	2.59 ± 0.041
TMB7	17.23 ± 0.22	12.1 ± 0.51	+++	237%	177%	1.5	1.4	1.83 ± 0.041
TMB8	16.9 ± 0.33	3 ± 0.18	+	0	0	0	0	1.55 ± 0.032
TMB9	13.8 ± 0.68	4.6 ± 0.27	+++	233%	140%	0	0	1.41 ± 0.052
TMB10	11.2 ± 0.6	0.3 ± 0.33	++	0	0	0	0	0.96 ± 0.02
TMB11	0	0	+	0	0	0	0	0.51 ± 0.029
TMB12	0	0	+	0	0	0	0	0
TMB13	0	2.8 ± 0.12	0	0	0	0	0	0

IAA was quantified by spectrophotometric pink coloration estimation method. Ten isolates, TMB1, TMB2, TMB3, TMB5, TMB6, TMB7, TMB8, TMB9, TMB10 and TMB13, showed IAA production ranging from 0.3–12.1 μg mL^−1^ at 2% NaCl supplementation. TMB7 showed the highest production of IAA, whereas TMB10 showed the lowest production of IAA (Table 2). IAA is a phytohormone also produced by many bacteria, which is involved in cell division, cell enlargement and root elongation (Bhutani et al., 2018b). Our results are in agreement with Widowati and Sukiman (2019), who reported IAA production up to 12.28 μg mL^−1^ in the endophytic bacteria of mungbean. Saleem et al. (2021) also demonstrated the mitigating efficiency of IAA production from salt tolerant bacteria isolated from cotton. Jabborova et al. (2020) also reported that endophytic bacteria have ability to produce IAA. Recently, Desai et al. (2023) explained the ability of salt-tolerant PGPB isolated from mungbean to produce IAA under salt stress. IAA production is one of the very important features for the screening of plant beneficial bacteria.

Nitrogen fixation ability of mungbean root endophytic bacteria was tested by growing bacteria on NFM agar plates. All isolates showed nitrogen fixation ability except TMB13 under saline conditions. TMB2, TMB7 and TMB9 showed highest ability (Table 2). Nitrogen is the most important element for plant growth and development. Bacteria produce nitrogenase enzyme to fix the atmospheric nitrogen (Gu et al., 2018). Favero et al. (2021a) isolated the nodule endophytic bacteria from the mungbean, which showed nodule formation and nitrogen fixation capability. *Bradyrhizobium* sp. exhibited the maximum potential for nodulation and nitrogen fixation. Tang et al. (2020) also reported that endophytic bacteria have the potential to fix biological nitrogen in tropical forest soil. Zhang et al. (2022) also identified that endophytic bacteria isolated from cassava roots exhibit nitrogen fixation ability. Potential root-associated bacteria can fix atmospheric nitrogen and alleviate nutrient stress in plants.

Zinc is an essential micronutrient involved in several cellular processes including metabolism, mitochondrial activity mitosis and cell development. It mainly participates in the redox reactions and works as a catalyst for enzymes (Ditta et al., 2022). Out of 13 root endophytic bacteria of mungbean, only three isolates, TMB6, TMB7 and TMB9, were able to solubilize zinc on tris minimal media supplemented with zinc oxide and zinc carbonate under saline conditions. TMB6 showed higher solubilization efficiency of 260% in zinc oxide and 200% in zinc carbonate media (Table 2). Previously, Singh et al. (2020) explained the zinc solubilizing ability of *Burkholderia arboris* and demonstrated the positive role of its inoculation in mungbean cultivation. Zinc solubilizing potential of bacteria was reported by several studies, which play crucial roles in soil fertility (Rani et al., 2022; Verma et al., 2022). Similarly, Ali et al. (2022) also demonstrated that endophytic bacteria have the potential to solubilize zinc and their combination with synthetic fertilizer significantly increased the plant growth compared to the sole application of chemical fertilizer.

Cellulase and pectinase activity of mungbean rhizobacteria was observed on NaCl supplemented plates. Out of 13 isolates, only TMB2, TMB6 and TMB7 showed cellulase activity, where TMB6 showed highest activity with 1.75 index. Only two isolates, TMB2 and TMB7, exhibited pectinase activity (Table 2). Cellulase and pectinase belongs to the family of hydrolytic enzymes. Hydrolytic enzymes play a pivotal role in the decomposition of dead organic matter present in the soil and provide nutrients to plants (Reetha et al., 2014). Recently, Reddy et al. (2022) reported PGPB have the ability to produce hydrolytic enzymes. Bhutani et al. (2021) also isolated cellulase and pectinase producing bacteria from the mungbean endosphere, which showed plant growth-promoting (PGP) potential. Dogan and Taskin (2021) also demonstrated that endophytic bacteria isolated from *Poaceae* plant displayed cellulase and pectinase production ability. Borah et al. (2019) also reported that endophytic bacteria from tea plant exhibit cellulase and pectinase activity. Cellulase and pectinase might enable bacteria to invade the roots and nodules of host plant. Futuristic comprehensive studies should be designed to explore the role of hydrolytic enzymes in root/nodule invasion and plant growth promotion by developing cellulase and pectinase negative mutants or using other cutting-edge techniques.

Biofilm formation activity was examined in microtiter plate assay. All bacterial isolates except TMB12 and TMB13 showed biofilm formation ranging 0.11–2.59 at OD_570nm_. TMB6 showed the highest efficiency of biofilm formation, while TMB1 showed the lowest efficiency of biofilm formation (Table 2). Several PGPB can effectively interact with the plants root zone and form biofilm on its surface, which protects plants against environmental stresses (Ansari and Ahmad, 2018). Previously, Yasmeen et al. (2020) isolated halotolerant bacteria from saline soil and demonstrated their biofilm formation ability under salt stress. Alaa (2018) also reported that *Pseudomonas anguilliseptica* have biofilm formation potential under different levels of salts. Generally, efficient biofilm forming bacteria perform their inherent functions effectively, even in the challenging environment (Tariq et al., 2014).

**Figure 2:**
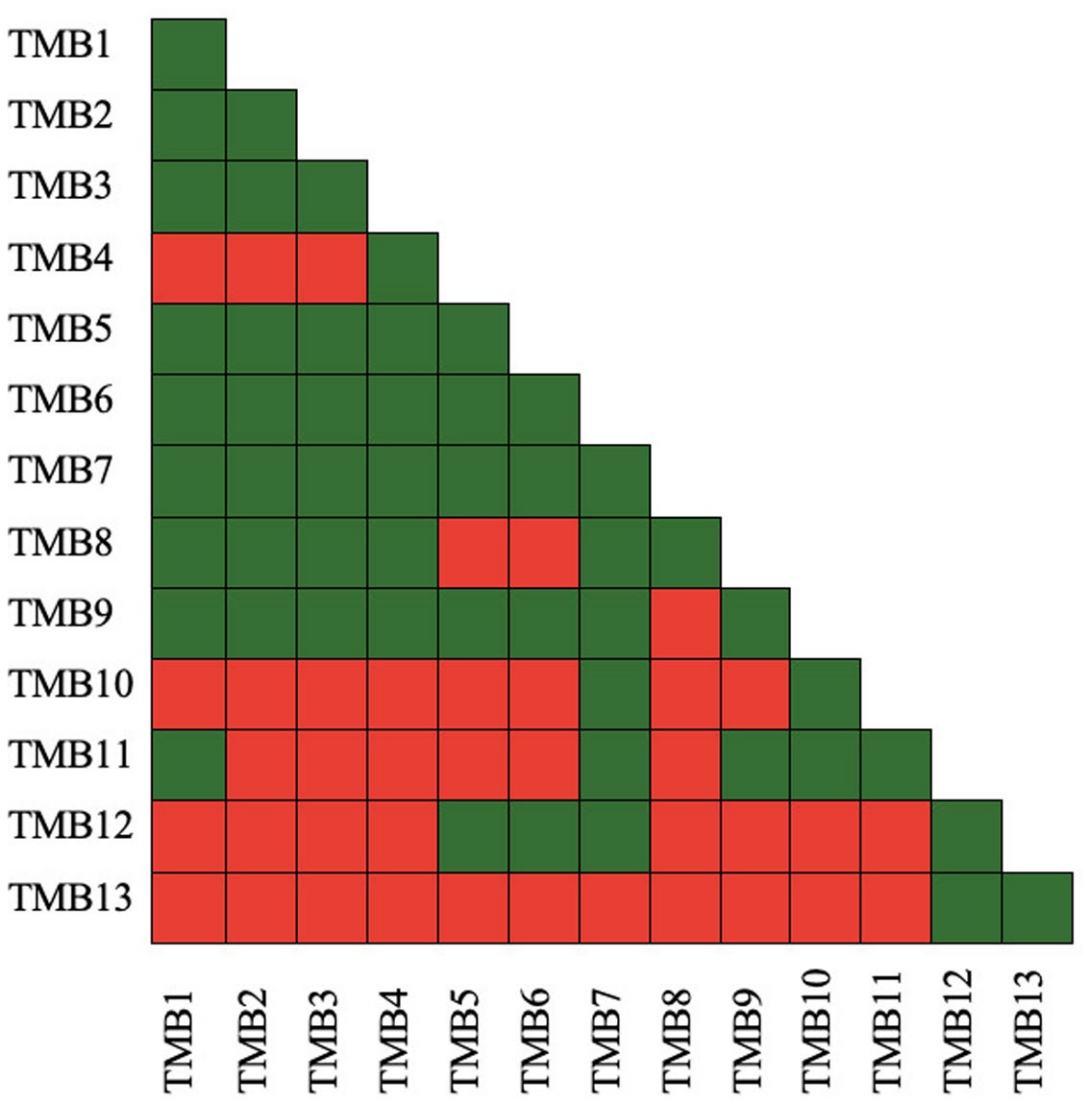
Antibiosis activity of mungbean root endophytic bacteria. Green color presents bacterial compatibility to grow together, while red color presents inhibitory interaction between bacteria. TMB7 demonstrated growth compatibility with most of the bacterial isolates, while TMB13 demonstrated growth inhibitory interaction with most of the bacterial isolates.

Antibiosis activity of isolates was checked by growing pair of bacteria together in an overlay plate assay. Bacterial isolates, TMB1, TMB2, TMB3, TMB5, TMB6, TMB7, TMB8 and TMB9, displayed maximum compatibility to grow together. TMB7 demonstrated the highest compatibility with all isolates except TMB13 (Figure 2). Kumawat et al. (2021) demonstrated that *Rhizobium* sp. and *Enterococcus mundtii* have growth compatibility. When these bacteria applied in consortia on mungbean the growth parameters of mungbean were increased as compared to single inoculation. Latha et al. (2009) also isolated *P. fluorescens* (Pf1 and Py15) and *B. subtilis* (Bs16) from tomato and demonstrated that all of three bacterial strains are compatible to grow together. Similarly, Ashraf et al. (2019) isolated four plant growth-promoting rhizobacteria (PGPR) isolates from wheat rhizosphere and checked their antimicrobial activity against three bacterial strains *Vibrio cholera*, *Enterobacter aerogenes,* and *Klebsiella pneumoniae.* Only one isolate showed antimicrobial activity against *K. pneumoniae* while others were compatible to each other. Compatible bacteria do not inhibit the growth of each other and perform effectively in consortium to promote plant growth.

### Phylogenetic identification of efficient STEB

**Figure 3:**
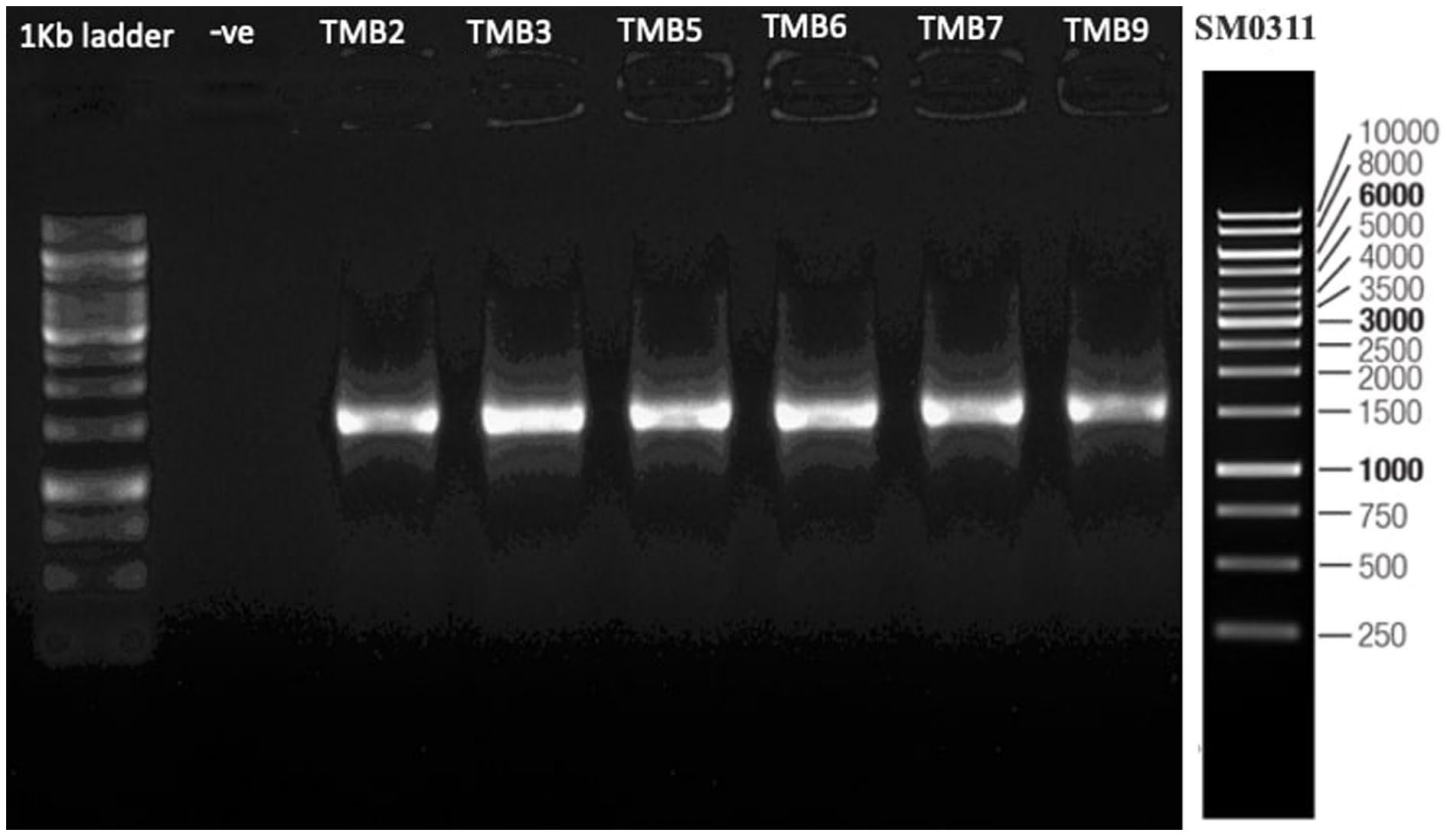
*16S rRNA* gene amplification of potential bacterial isolates. Amplification of DNA bands of 1,500 bp were produced, which were confirmed by comparing with 1 kb DNA ladder.

**Figure 4:**
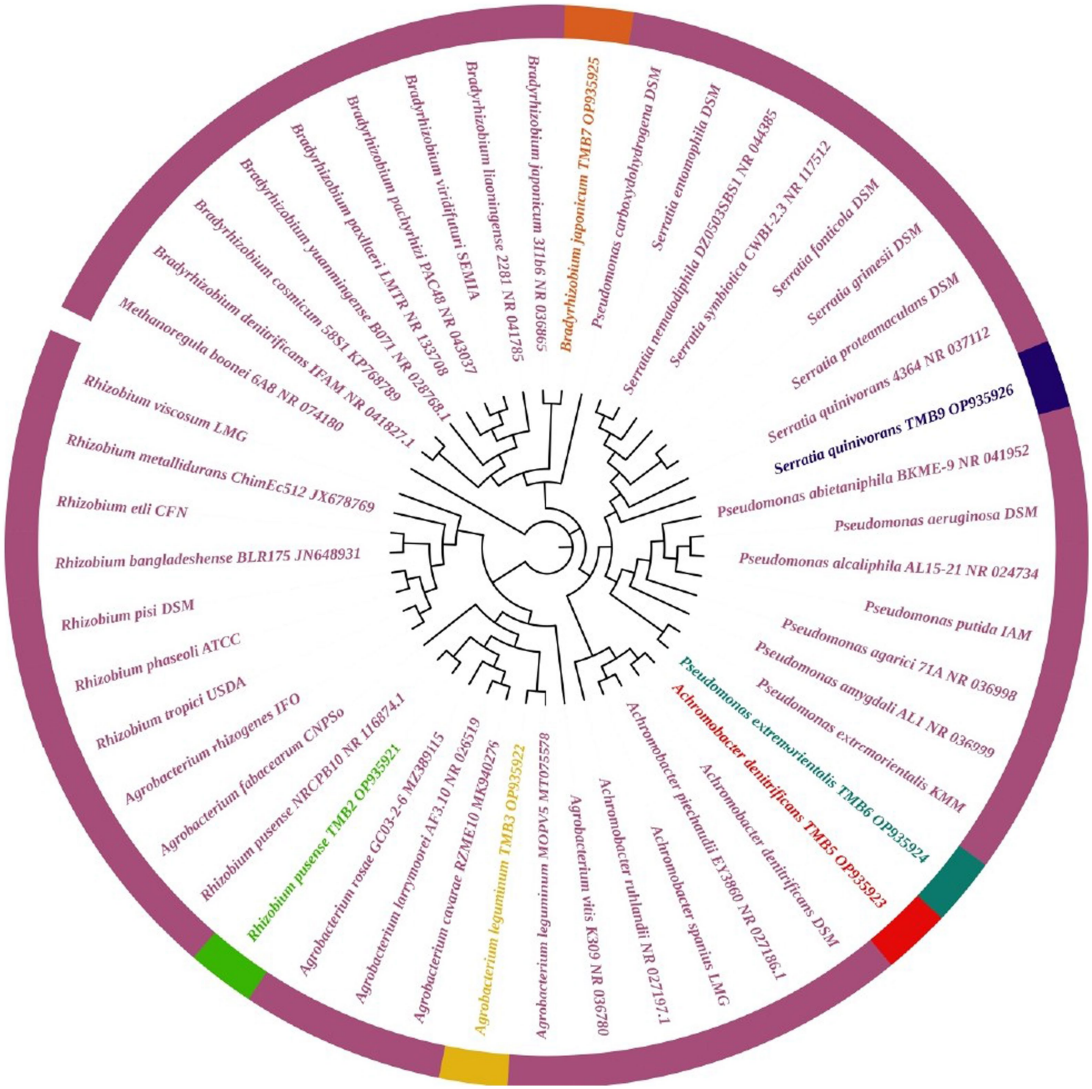
Phylogenetic tree of mungbean root endophytic bacteria. All the sequences were grouped into 3 clades. TMB2 positioned in the neighborhood of *Rhizobium pusense*, TMB3 in *Agrobacterium leguminum*, TMB5 in *Achromobacter denitrificans*, TMB6 in *Pseudomonas extremorientalis*, TMB7 in *Bradyrhizobium japonicum* and TMB9 in *Serratia quinivorans*.

**Figure 5:**
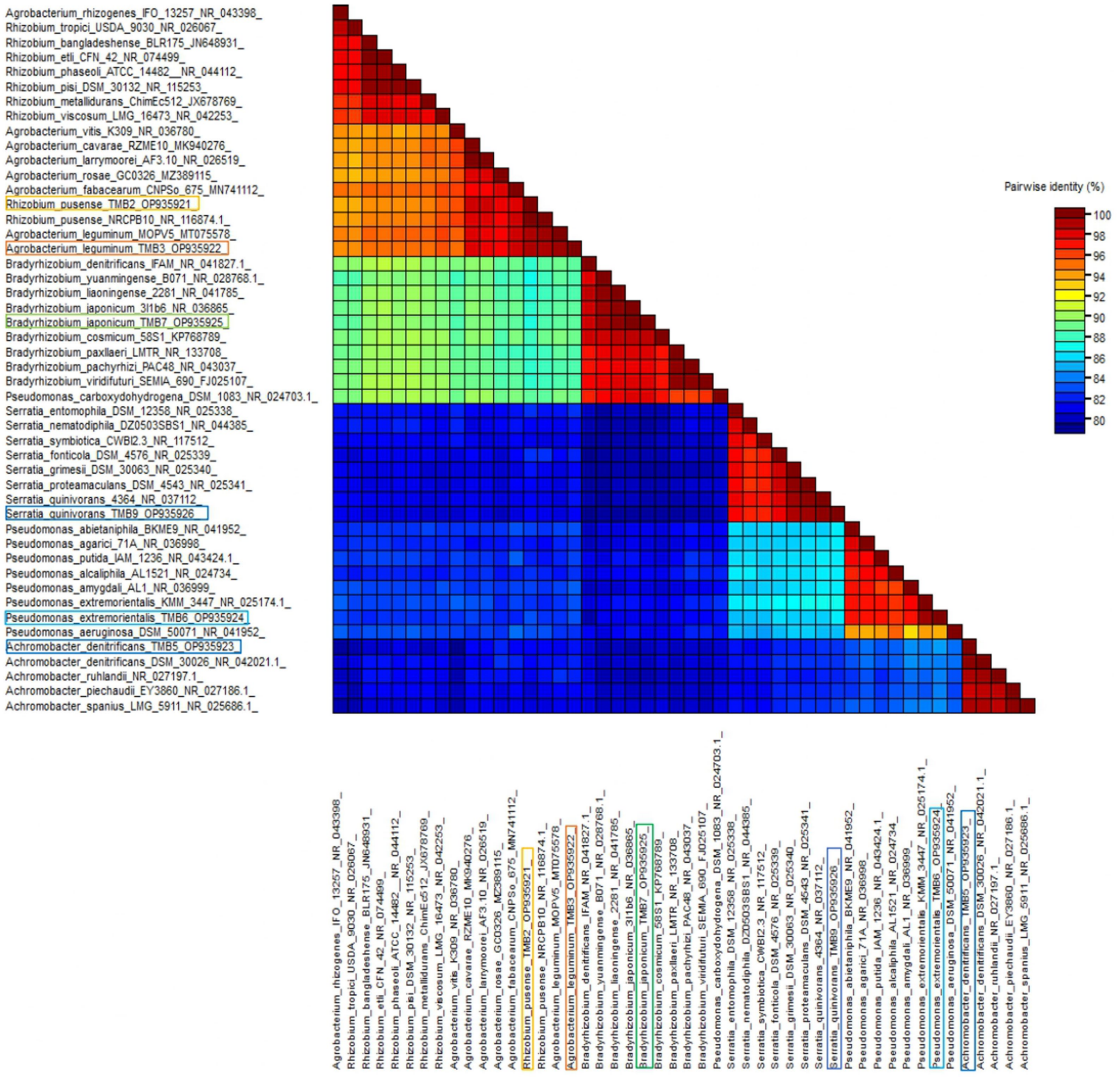
Pairwise identity chart of mungbean root endophytic bacteria. The identity percentage of different bacterial sequences ranged 80–100.

Amplification of *16S rRNA* gene using fD1 and rD1 primers produced approximately 1,500 bp DNA band as shown in Figure 3. After sequencing and assembling, DNA sequence contigs of more than 1,400 nt were generated. Sequences of *16S rRNA* showed maximum similarity of more than 98% with the different sequences available in nucleotide databases and identified TMB2 as *Rhizobium pusense*, TMB3 as *Agrobacterium leguminum*, TMB5 as *Achromobacter denitrificans*, TMB6 as *Pseudomonas extremorientalis*, TMB7 as *Bradyrhizobium japonicum* and TMB9 as *Serratia quinivorans*. Sequences were deposited in NCBI GenBank under the accession numbers OP935921–OP935926 (Table 3). Phylogenetic tree of these sequences was constructed with 42 authentic sequences belonging to 6 identified genera using maximum likelihood method with 1,000 bootstrap value and *Methanoregula boonei* was used as outgroup. All the sequences were grouped into 3 clades belonging to common ancestor. TMB7 was placed in clade 1, TMB6, TMB5 and TMB9 in clade 2 and TMB2 and TMB3 in clade 3 shown in Figure 4. A color coded pairwise identity matrix was also created, in which each colored cell represents the percentage identity of two sequences. The identity percentages between the selected sequences were ranging 80–100 (Figure 5).

**Table 3:** Taxonomic identification of potential salinity-tolerant endophytic bacteria.

Bacterial isolates	Taxonomic identification	Percentage identity	Accession numbers
TMB2	*Rhizobium pusense*	98.81%	OP935921
TMB3	*Agrobacterium leguminum*	99.79%	OP935922
TMB5	*Achromobacter denitrificans*	99.17%	OP935923
TMB6	*Pseudomonas extremorientalis*	98.93%	OP935924
TMB7	*Bradyrhizobium japonicum*	100%	OP935925
TMB9	*Serratia quinivorans*	98.30%	OP935926

Existence of *Rhizobium pusense* and *Bradyrhizobium japonicum* in mungbean root has been reported in the literature. *Rhizobium pusense* colonize mungbean roots and improve plant growth by producing phytohormones (Chaudhary et al., 2021). Similarly, Nguyen et al. (2022) demonstrated the occurrence of *Rhizobium pusense* in rice and increased its growth and yield upon inoculation. Members of genus *Bradyrhizobium* dominantly exist in the roots and nodules of mungbean and soybean. Favero et al. (2021b) isolated *Bradyrhizobium japonicum* from mungbean nodules and demonstrated the positive effect on yield and growth of mungbean plant. Yasmeen et al. (2012) also explained that occurrence of *Bradyrhizobium japonicum* in mungbean. Similarly, Chhetri et al. (2019) isolated *Bradyrhizobium japonicum* from root nodules of soybean. Generally, bacteria belonging to *Rhizobia* are well-known for nitrogen fixation, which ultimately increases crop yield (Anjum et al., 2006).

In this study, we reported the occurrence of *Agrobacterium leguminum, Achromobacter denitrificans*, *Pseudomonas extremorientalis* and *Serratia quinivorans* in the roots of mungbean for the first time. Recently, Castellano-Hinojosa et al. (2021) isolated *A. leguminum* from the *Phaseolus vulgaris* nodules and claimed it as a novel species based on the data obtained from colony morphology, sequence analysis, phylogenetic analysis and taxonomic characterization. Previously, Sultana et al. (2020) isolated *A. denitrificans* from the rice plant, which showed PGP properties under salt stress. Wang et al. (2019) isolated *P. extremorientalis* from the rhizosphere of pear plant. Kaur et al. (2022) reported the existence of *P. extremorientalis* in the endophytic region of wheat*. P. extremorientalis* improved plant growth under salt stress by reducing harmful effects of salt (Egamberdieva et al., 2016). Recently, researchers have revealed the existence of *S. quinivorans* in the oak, *Petroselinum crispum* and *Picrorhiza kurroa* (Kumar et al., 2021; Reis et al., 2021; Chlebek et al., 2022). Novel plant-bacterial associations might be due to the changes in environmental conditions.

### Biofertilizers potential of STEB under controlled-conditions

**Figure 6:**
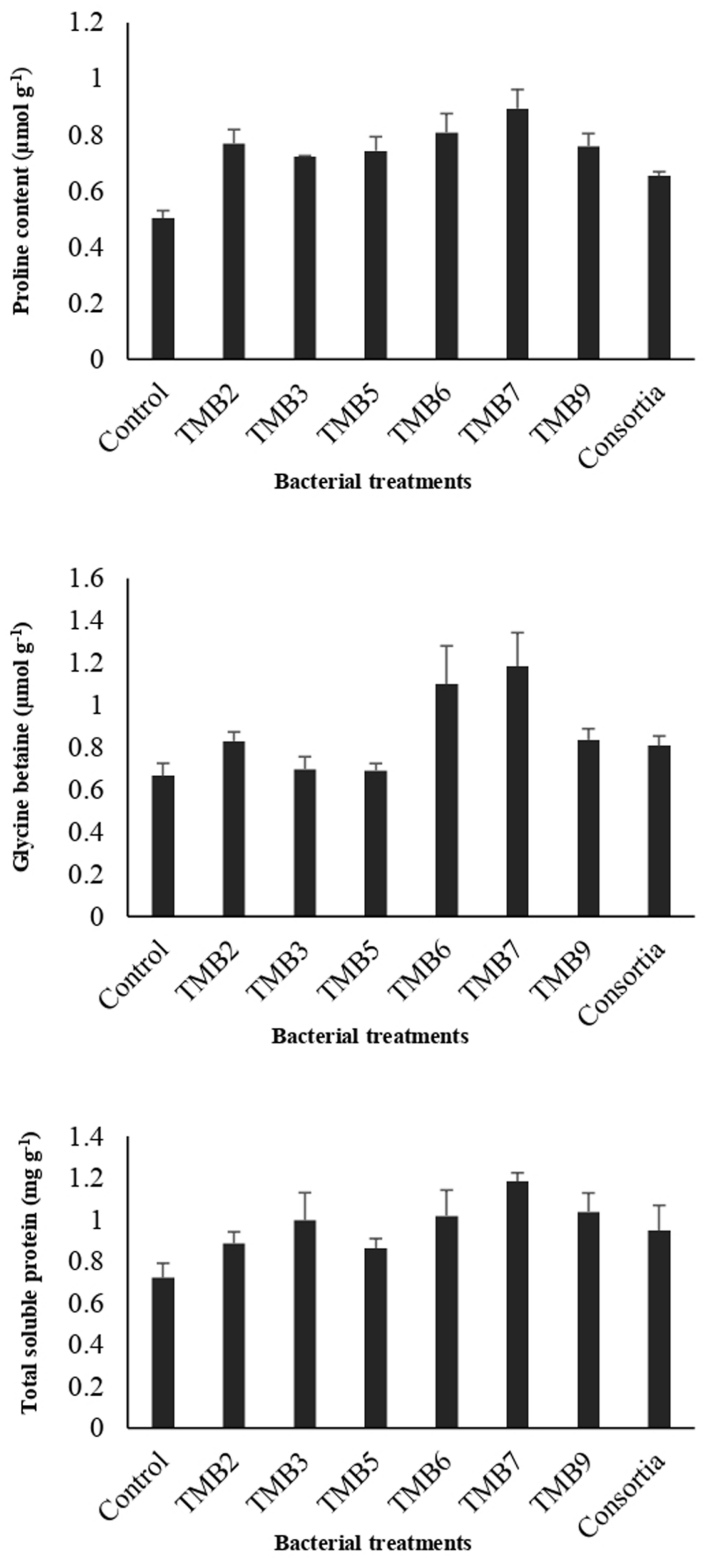
Effect of potential salt-tolerant endophytic bacteria (STEB) on biochemical contents of mungbean under salt stress. Concentrations of proline, glycine betaine and total soluble proteins were determined. Inoculation of TMB7 showed significant potential to increase proline contents by 77%, glycine betaine by 78% and total soluble proteins by 64% compared to control.

Potential isolates including TMB2, TMB3, TMB5, TMB6, TMB7, TMB9 and consortia were tested for PGP properties under controlled-conditions experiment (Supplementary Figure S1). After 6 weeks of inoculation, agronomical parameters were calculated and statistically analyzed (Table 4). Inoculation of bacterial isolates, *P. extremorientalis* TMB6, *B. japonicum* TMB7 and *S. quinivorans* TMB9, showed maximum potential in improving plant growth parameters. TMB2, TMB6 and TMB7 showed a significant increase in root length compared to control. All isolates exhibited a significant increase in shoot length compared to control except consortia. TMB6 and TMB7 showed a significant increase in plant fresh weight. *P. extremorientalis* TMB6, *B. japonicum* TMB7 and *S. quinivorans* TMB9 were most efficient and showed a significant increase in plant dry weight by 52, 61 and 45%, respectively, compared to control. Nodulation was observed by the inoculation of TMB2, TMB7 and consortia. Inoculation of *B. japonicum* TMB7 showed maximum potential to increase plant growth parameters, i.e., root length (59%), shoot length (45%), fresh weight (67%) and dry weight (61%) among all isolates. Consortia did not show any positive effect on plant growth. Biochemical attributes, i.e., proline content, glycine betaine and total soluble proteins were increased by all treatments of root endophytic bacteria under salt stress as shown in Figure 6. Inoculation of TMB7 showed significant potential to increase proline contents by 77%, glycine betaine by 78% and total soluble proteins by 64% compared to control.

**Table 4:** Effect of potential salinity-tolerant endophytic bacteria on mungbean growth under controlled-conditions.

Treatment	Root length (cm)	Shoot length (cm)	Plant fresh weight (mg)	Plant dry weight (mg)	Number of nodules per plant
Control	21 ± 1.7^cd^	30.8 ± 1.3^b^	737 ± 10^e^	82.5 ± 8^cd^	0
TMB2	29.5 ± 1.32^a^	42.8 ± 1.6^a^	950 ± 30^d^	110 ± 20^abc^	26 ± 0.8^b^
TMB3	29.3 ± 1.4^ab^	42 ± 2.0^a^	920 ± 60^d^	90 ± 8^bcd^	0
TMB5	22 ± 1.5^cd^	41.5 ± 1.7^a^	970 ± 50^cd^	105 ± 20^abcd^	0
TMB6	31.5 ± 1.3^a^	43 ± 1.1^a^	1,160 ± 40^ab^	125 ± 6^ab^	0
TMB7	33.3 ± 1.8^a^	44.8 ± 0.9^a^	1,230 ± 20^a^	132.5 ± 8^a^	31.3 ± 0.5^a^
TMB9	24.8 ± 1.1^bc^	42.5 ± 0.6^a^	1,080 ± 40^bc^	120 ± 12^ab^	0
Consortia	19 ± 2.1^d^	32.3 ± 0.9^b^	640 ± 20^e^	72.5 ± 6^c^	19.5 ± 1^c^
LSD (0.05)	4.53	3.9	117	37	1.4
ANOVA	***	***	***	*	***

Each value represents mean (*n* = 4) ± standard error. Values followed by the different letters in same column indicate significant difference and followed by same letters are not significantly different. ^*^Represents significance and ^***^ represents high significance. LSD, least significant difference.

PGPB have the ability to enhance plant biochemical attributes such as proline, glycine betaine and total soluble proteins under salt stress to overcome the effects of salinity on plant growth. Proline and glycine betaine play an important role as osmoprotectants and osmoregulatory elements to reduce the harmful effects of salinity by boosting the defense mechanism against oxidative damage under salt stress conditions (Diagne et al., 2020). Our results are in agreement with the previous studies which reported that the inoculations of bacteria enhanced legumes plant biochemical properties such as proline and glycine betaine content under salt stress (Ashraf and Bashir, 2003). Irshad et al. (2021) demonstrated that inoculation of *Rhizobium* sp. enhanced salt tolerance in *medicago truncatula* by increasing glycine betaine, proline, total soluble proteins and solutes contents. Mushtaq et al. (2021) also revealed that inoculation of *Rhizobium* enhanced total soluble proteins and proline contents in *Cicer arietinum* to alleviate salt stress.

Kaur et al. (2022) described that the inoculation of *P. extremorientalis* on peal millet increased plant growth parameters which are in agreement with our results. Devi et al. (2022) also demonstrated that inoculation of *P. extremorientalis* increased the plant growth parameters such as fresh weight, dry weight, shoot length and root length of chili. Similarly, Kiruthika and Arunkumar (2021) also demonstrated that inoculation of *B. japonicum* increased fresh and dry weight of mungbean. Miljaković et al. (2022) also reported that inoculation of *B. japonicum* improved growth parameters of soybean. Similarly, Zveushe et al. (2023) demonstrated that *Bradyrhizobium japonicum* enhanced plant growth parameters of soybean under salt stress. Our results are in agreement with Kumar et al. (2021), who demonstrated the potential role of *S. quinivorans* inoculation to increase the growth of *Picrorhiza kurroa* under control condition experiments. In this study, consortia did not perform well for plant growth promotion. Our results are in disagreement with Mogal et al. (2022) who reported that consortia of rhizobial bacteria have positive effects on plant growth parameters of mungbean. Previously, Consentino et al. (2022) described that inoculation of consortia did not improve growth parameters of lettuce, compared to the inoculation of pure bacterial culture. Poor performance of bacterial consortia can be attributed to the antagonism which may exist among the different bacteria of consortia. Single inoculations performed better for mungbean growth promotion and the extent of growth improvement corresponds to the bacterial ability to produce plant growth-promoting substances.

## Conclusion

Out of thirteen root endophytic bacteria, six isolates, TMB2, TMB3, TMB5, TMB6, TMB7 and TMB9, were able to tolerate salinity up to 2% NaCl and have the *in vitro* potential to produce plant growth-promoting substances under salt stress conditions. Phylogenetic analysis revealed the novel association of *Agrobacterium leguminum, Achromobacter denitrificans, Pseudomonas extremorientalis* and *Serratia quinivorans* with roots of mungbean. Inoculation of bacterial isolates, *Pseudomonas extremorientalis* TMB6, *Bradyrhizobium japonicum* TMB7 and *Serratia quinivorans* TMB9, showed maximum potential in improving plant growth and development under salt stress conditions. These potential salt-tolerant endophytic bacteria can be used as biofertilizer after field-testing for better production of mungbean crop at salt-affected lands.

## Data availability statement

The datasets presented in this study can be found in online repositories. The names of the repository/repositories and accession number(s) can be found in the article/Supplementary material.

## Author contributions

MT and SZ conceived the project and designed the study. SZ, MT, AA, and MA collected the samples and performed the experiments. SZ, MT, MA, MZ, and MS performed data analysis. SZ, MT, MA, MZ, and TY wrote the manuscript. SZ, MT, TY, HZ, and MA applied statistics and critically reviewed manuscript. All authors contributed to the article and approved the submitted version.

## Supplementary material

### Supplementary Figure

Effect of inoculation of potential salt-tolerant endophytic bacteria (STEB) on mungbean growth under controlled-conditions. Inoculation of bacterial isolates, *P. extremorientalis* TMB6, *B. japonicum* TMB7 and *S. quinivorans* TMB9, showed maximum potential in improving plant growth parameters. Photographed at 4-week stage.
