# Supplementary figures and images for: Probing the potential of salinity-tolerant endophytic bacteria to improve the growth of mungbean [Vigna radiata (L.) Wilczek]
Source: Front Microbiol. 2023 Dec 4;14:1149004. doi: 10.3389/fmicb.2023.1149004 (PMC10725929; doi:10.3389/fmicb.2023.1149004)

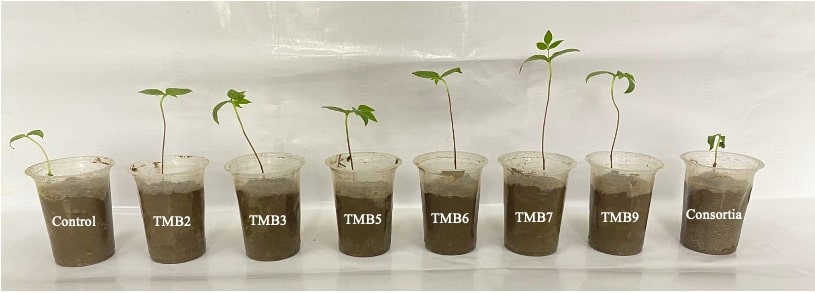

Supplement: Supplementary file 2 [file Image_1.JPEG]
